# Multimodality imaging of a cardiac paraganglioma: A case report

**DOI:** 10.3389/fcvm.2023.1123789

**Published:** 2023-03-24

**Authors:** Bruna Punzo, Dario Baldi, Brigida Ranieri, Carlo Cavaliere, Filippo Cademartiri

**Affiliations:** ^1^ IRCCS SYNLAB SDN, Naples, Italy; ^2^ Department of Radiology, Fondazione Toscana Gabriele Monasterio/CNR, Pisa, Italy

**Keywords:** case report, cardiac paraganglioma, neuroendocrine tumor, CCT, CMR

## Abstract

Cardiac paragangliomas (PGLs) are rare extra-adrenal tumors that arise from chromaffin cells of the sympathetic ganglia. PGLs are often diagnosed incidentally, in the absence of symptoms, or with symptoms related to cardiovascular dysfunction. Cardiac computed tomography (CCT) and cardiac magnetic resonance (CMR) can be used to accurately determine the lesion morphology and position as well as providing detailed tissue characterization. A multimodal imaging approach, not yet standardized, could be useful either in diagnosis and monitoring or in treatment planning. In the case reported here, CCT and CMR were performed to define lesion anatomy, and a reconstruction was generated using cinematic rendering (CR) to characterize the PGL angioarchitecture.

## Introduction

 According to the World Health Organization’s tumor classification scheme, paragangliomas (PGLs) are rare neuroendocrine tumors arising from extra-adrenal parasympathetic or sympathetic ganglia neural crest cells (1).

Cardiac PGLs are extremely rare tumors (<1% of all primary cardiac tumors) originating from visceral paraganglial cells of the left atrium or the aorta. Cardiac PGLs are most commonly observed in the left atrium and they are functional in 35%–50% of patients with symptoms related to catecholamine excess (2, 3). Clinically, these patients may present without symptoms or with generalized disorders such as hypertension, dyspnea, or cardiovascular risk factors.

These lesions can be evolutive, leading to serious complications such as bleeding and cardiac failure. Radiologic findings acquired by computed tomography (CT) and magnetic resonance (MR) play an essential role in cardiac PGL management and can provide further anatomic and tissue characterization.

Currently, the most commonly used tool to provide 3D images from CT data is volume rendering (VR). The cinematic rendering (CR) algorithm has recently been introduced; this supports interpretation for diagnosis of various cardiac pathologies and treatment planning by creating complex lighting effects such as refraction, providing high levels of detail in terms of shadowing and depth, and visualizing high-density and high-contrast structures (4).

In this study, we report the case of a patient with severe rest dyspnea, on whom cardiac computed tomography (CCT) and cardiac magnetic resonance (CMR) imaging were performed to characterize a vascularized paracardiac mass suggested by a previous invasive coronary angiography (ICA) addressing a possible angioma.

CR reconstruction using the CCT dataset was employed to better evaluate the anatomical details, including course and tortuosity of the vessels (e.g., coronaries, cardiac veins, and large vessels) involved in the mass vascular supply.

## Case description

**Figure 1:**
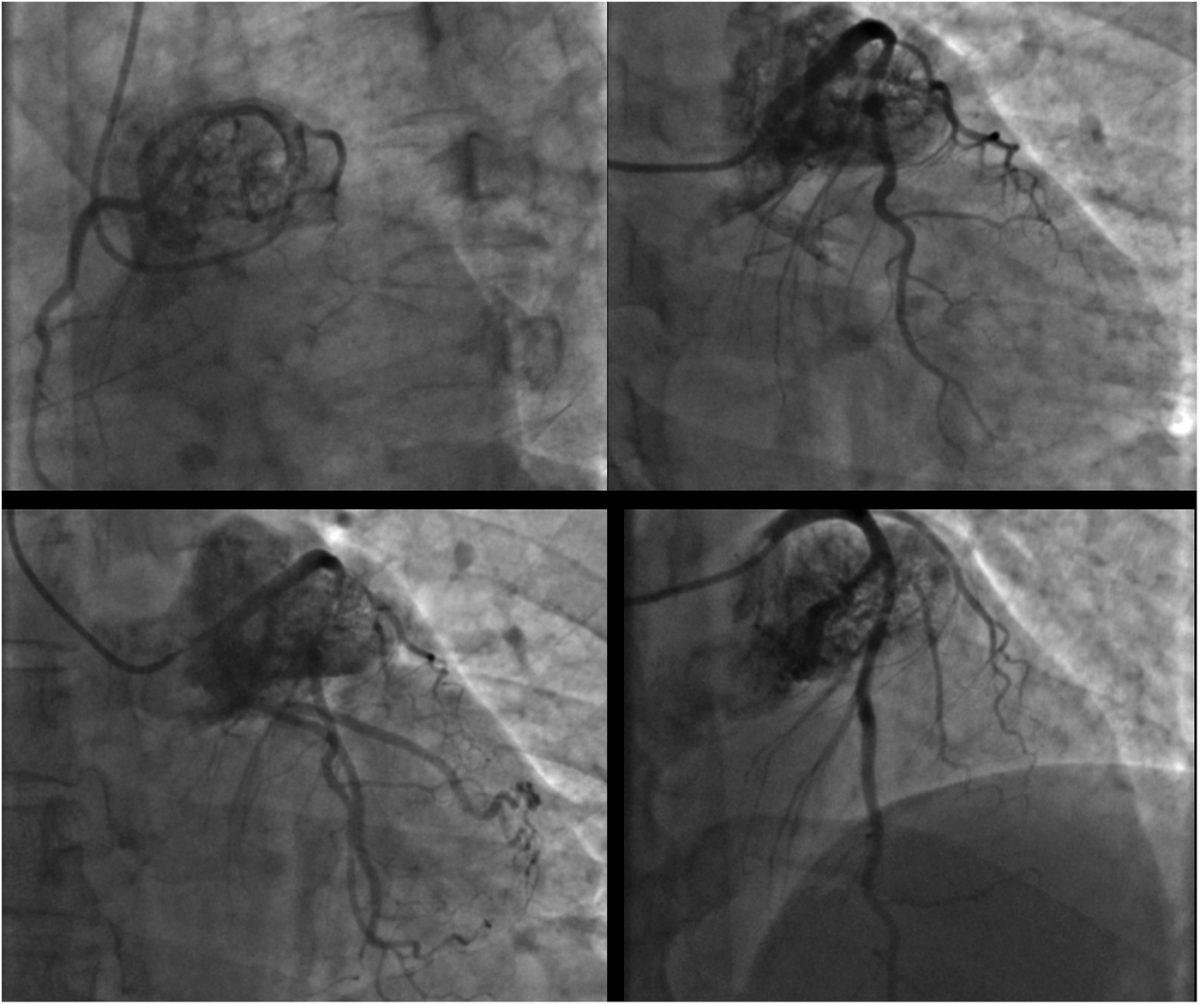
Coronary angiogram in different oblique projections showing a large cardiac mass encasing the left coronary artery.

A 46-year-old man, a candidate for aortic valve replacement, was referred to us after an invasive coronary angiogram (ICA; Figure 1; Supplementary Movie 1) showed a large cardiac mass encasing the left coronary artery; there was no coronary artery stenosis. The patient presented with a family history of atherosclerotic coronary artery disease, hypertension treated with lecardipine and lobizide, type 2 insulin-dependent diabetes mellitus, and obesity (BSA = 2.10 m^2^). The patient provided written informed consent for this study.

**Figure 2:**
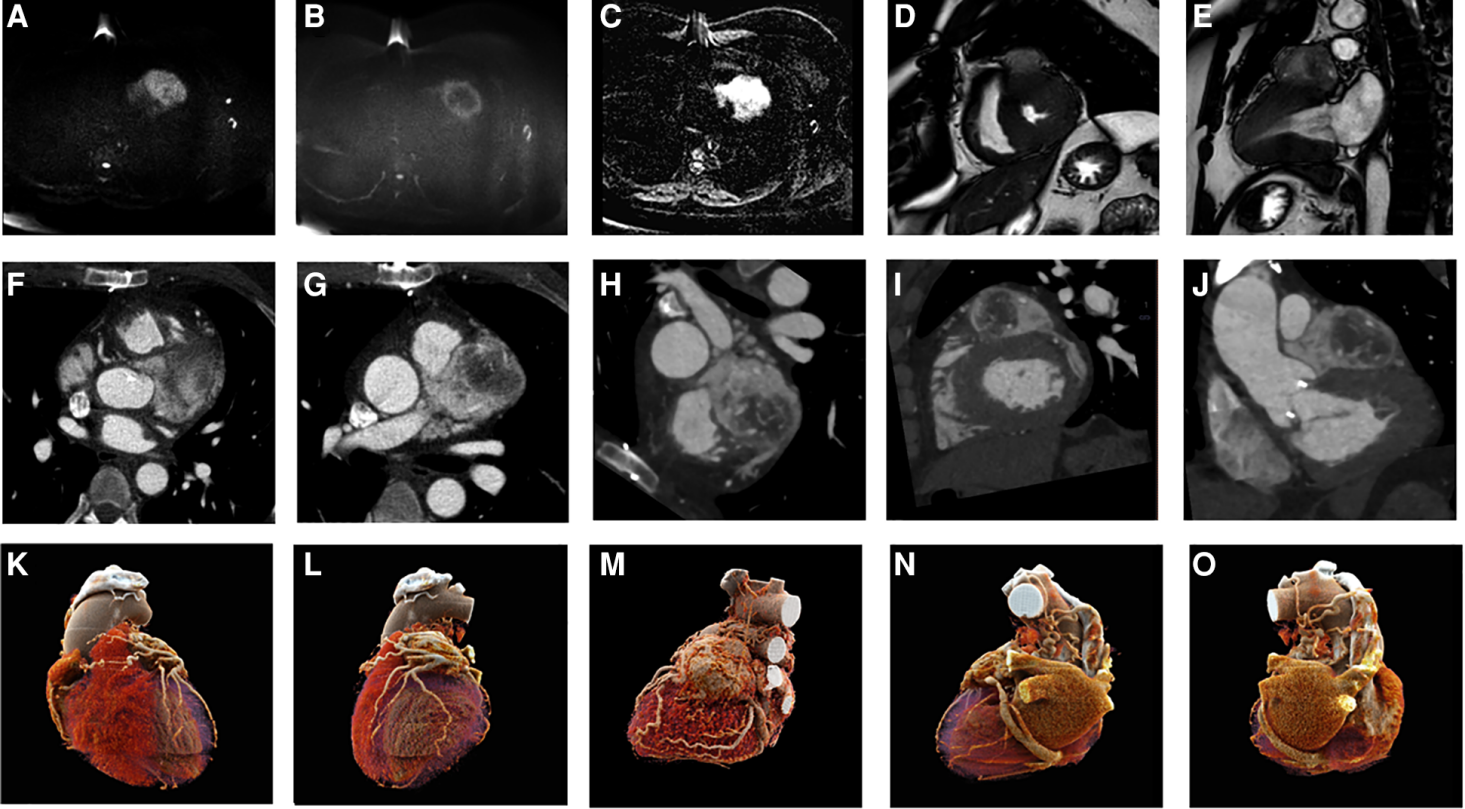
CMR acquisition (**A–E**): DWI b 0 and b 1,000 (**A,B**) and ADC map (**C**) showing high cellularity of the lesion; balanced gradient echo sequence in short axis and two chambers (**D,E**), showing morphology and extension of a PGL. CCT acquisition (**F–J**): axial (**F,G**) and oblique planes highlighting the morphology, position, and contrast of the PGL. CR reconstructions in different planes (**K–O**).

CCT was performed (Figures 2F–J; Supplementary Movies 2 and 3) using a third-generation dual source CT scanner (DSCT; Somatom Force, Siemens Healthineers, Erlangen, Germany). Initially, a non-contrast CT prospectively ECG-triggered high-pitch spiral acquisition was performed for calcium score evaluation. Subsequently, an angiographic CT scan with retrospective ECG gating and automated attenuation-based anatomical tube current modulation (CARE Dose 4D, Siemens) was acquired. Tube voltage was adjusted by using the automated attenuation-based tube voltage selection functionality (CAREkV, Siemens). For the angiographic scan, 70 mL of iodinated contrast agent (Iomeprol 400 mgI/mL, Iomeron 400, Bracco, Italy) was injected at 5.5 mL/s, followed by 50 mL of saline at the same flow rate. Data were reconstructed using a dedicated third-generation Advanced Modeled Iterative Reconstruction device (ADMIRE, Siemens Healthineers, Erlangen, Germany) using medium-sharp convolution kernels (Bv36 and Bv40), a strength level of 3, and a section thickness of 0.75 mm with an increment of 0.4 mm and a pixel matrix of 512 × 512. Postprocessing was performed using a dedicated workstation (Syngo.Via VB10B, Siemens Healthineers, Erlangen, Germany), and MIP, c-MPR, 3D volume rendering, and cinematic rendering images were generated.

CCT showed a solid mass above the basal anterior wall of the left ventricle, characterized by intense, progressive, and inhomogeneous contrast enhancement, and a capsule; the core of the lesion was hypovascular/necrotic; in addition, the lesion showed complete encasing of the left main coronary artery, the proximal-middle segment of the left anterior descending (LAD) coronary artery, and the proximal segment of the left circumflex coronary artery. CR reconstruction of the CCT dataset was employed to better evaluate the anatomical details involved in the mass vascular supply.

The patient also underwent CMR imaging (Figures 2A–E) on a 1.5 T scanner (Achieva dStream 1.5 T, Philips) with a 32-channel body coil. The CMR protocol consisted of cine sequences sBTFE in three cardiac planes (two and four chambers and short axis); T2 STIR and DWI of the entire left ventricle in the short-axis plane; tissue characterization sequences (T1 native MOLLI and T2* mapping); delayed enhancement PSIR-TFE for the evaluation of early or late gadolinium enhancement.

CMR findings were as follows: well-circumscribed ovoid/spherical lesions and typical hyperintensity on T2 imaging sequences, with the other features similar to CCT.

The location and evidence on the morphological, functional, and metabolic features of the mass all pointed to a solid, hypervascular, capsulated, non-infiltrative, neuro-endocrine type of tumor; even though serum catecholamines were within normal ranges, the diagnosis was non-excreting cardiac PGL.

The patient is now in follow-up, mainly with CMR for 2.5 years, and the morphological features of the mass are stable. Multimodality imaging of atypical cardiac masses is warranted for proper diagnosis and follow-up.

## Discussion

Extra-adrenal PGLs are solid hypervascularized tumors originating from the chromaffin cells of the sympathetic ganglia. PGLs are exceptionally rare (<1% of all cardiac primary tumors), and their diagnosis and treatment usually follow very different paths and approaches.

In our case, the mass is attached to the left upper anterolateral portion of the heart and incorporates an important tract of the left coronary artery as well as having contiguity relationships with the majority of adjacent structures.

These aspects, as also reported in the literature, make any surgical removal procedure extremely complex and risky, although the possibility of cardiovascular morbidity and mortality without treatment remains high, given the possible compression/invasion of neighboring structures.

Prognostic factors are represented by tumor attenuation, contrast enhancement, detailed understanding of the feeding vessel, and close proximity to surrounding blood vessels and vital structures (5, 6).

CCT and CMR, thanks to multiple 2D postprocessing techniques, are available to facilitate evaluation of the anatomy, which is often complex and challenging in patients with cardiac PGL.

CCT, thanks to its high spatial resolution and contrast, was able to evaluate the entire solid nodular mass at the base of the LV and its relationship with the surrounding arterial and venous vessels. The lesion was found to have stretched and shifted both the anterior descending coronary artery and the left circumflex artery, which, however, was not providing vascular supply.

In this context, to develop a clear understanding of the PGL angioarchitecture, CR reconstructions were generated.

Briefly, under this approach, 2D reconstructed slices composed of isotropic voxels from standard clinical CT acquisitions are stacked to create a volume, and light is passed through the volume to create a 3D visualization. However, compared to VR, CR makes use of a more sophisticated lighting model that creates photorealistic images with improved detail and produces shadowing effects that allow for robust visualization of the relative positions of structures.

3D visualizations of CT data can be invaluable in the investigation of complex anatomy and pathology, and pathological findings concerning neighboring structures can be identified and visualized (7).

3D CR is helpful for surgeons because it provides information on spatial relationships and a subjective perspective on the relevant organ (8).

Similar to VR, CR provides the best visualizations of high-density and high-contrast structures such as contrast-enhanced small vessels, but at the same time, it provides more natural and photorealistic illumination of the rendered data (9), highlighting relevant information in anatomically complex regions, as in congenital cardiopathies (10).

The applications of CR are numerous and varied, including medical education, easy disease detection, and improved description and classification of lesions. Recently, examples of the advantages of CR in the context of cardiovascular pathologies have been presented and this method has been qualitatively compared with other 3D postprocessing methods (11, 12). Moreover, realistic shadowing effects and different windowing options are likely to meet with more approval in imaging providing surgical and procedural guidance (13).

In our case, multiplanar reconstruction images still represented the gold standard in classification and preoperative treatment planning; however, the 3D volume rendering and cinematic rendering images provided a more exhaustive global overview of the vascular complexity of the lesion.

For morphological evaluation and tissue characterization, acquisition of a CMR scan is useful in the differential diagnosis and monitoring of cardiac masses. Through this investigation, the study of the cardiac planes enables localization of the lesion and determination of its relationship with the surrounding structures. The morphological characteristics of hyper/hypointensity are suitable to discriminate the lesion, and its relationships with other structures are much better highlighted.

In this case, the lesion showed obvious signal restriction in DWI sequence, and furthermore, the use of gadolinium (LGE) highlighted capsular hyperenhancement of the mass in post-contrast PSIR-TFE sequences, emphasizing its high vascularity.

CMR is now in routine use, but the examination process is lengthy, and it may not be available at every facility.

Both CCT and CMR are useful for further evaluation of these masses. Attenuation on CCT, tissue characterization on CMR, pattern and degree of contrast enhancement, and presence or absence of blood flow on cine CMR images can help differentiate among pericardial masses.

CMR provides incremental prognostic value over clinical factors such as left ventricular ejection fraction and coronary artery disease. In (14), CMR had high diagnostic accuracy in a patient with suspected cardiac tumors.

In our case, CMR contributed useful information, such as the morphological characteristics of hyper/hypointensity, signal restriction in DWI, and enhancement postgadolinium injection.

This case study shows the ability of a multimodal morphological imaging approach, using images obtained with CCT and CMR, to correctly identify, locate, and characterize cardiac PGLs and monitor non-operable lesions over time.

In challenging circumstances, extra-adrenal PGL and metastases can be localized using nuclear medicine methods such as SPECT and MIBG with neurotransmitter analogs, and metabolic radiotracers represent the final step for accurate functional characterization of a PGL and exclusion of a malignant component.

For this type of lesion with complex morphology, not only do additional visualization techniques such as CR provide descriptions and detailed representations of the lesion, its anatomy, and its relationship to the surrounding structures, but they also act as support for both diagnosis and management of complex cardiovascular lesions. In vitro analyses should be performed to characterize the functional status of PGLs, a feature that, combined with the vascular relationships of the lesion, deeply influences the management of these patients.

In conclusion, no standalone scans allow for comprehensive diagnosis of PGLs, although PET/MR technology and, more recently, radiomics techniques can attempt to solve issues related to morpho-functional assessment and benign/malign differentiation. In order to accomplish the aim of establishing the optimal ways to make use of these techniques, more studies with larger samples are needed.

## Data Availability

The datasets presented in this study can be found in online repositories. The names of the repository/repositories and accession number(s) can be found in the article/**Supplementary Material**.
