# Supplementary material for: Multimodality imaging of a cardiac paraganglioma: A case report
Source: Front Cardiovasc Med. 2023 Mar 24;10:1123789. doi: 10.3389/fcvm.2023.1123789 (PMC10080151; doi:10.3389/fcvm.2023.1123789)
Supplement: Supplementary file 1 [file Datasheet1.docx]

Supplementary Material

# Supplementary Figure 1


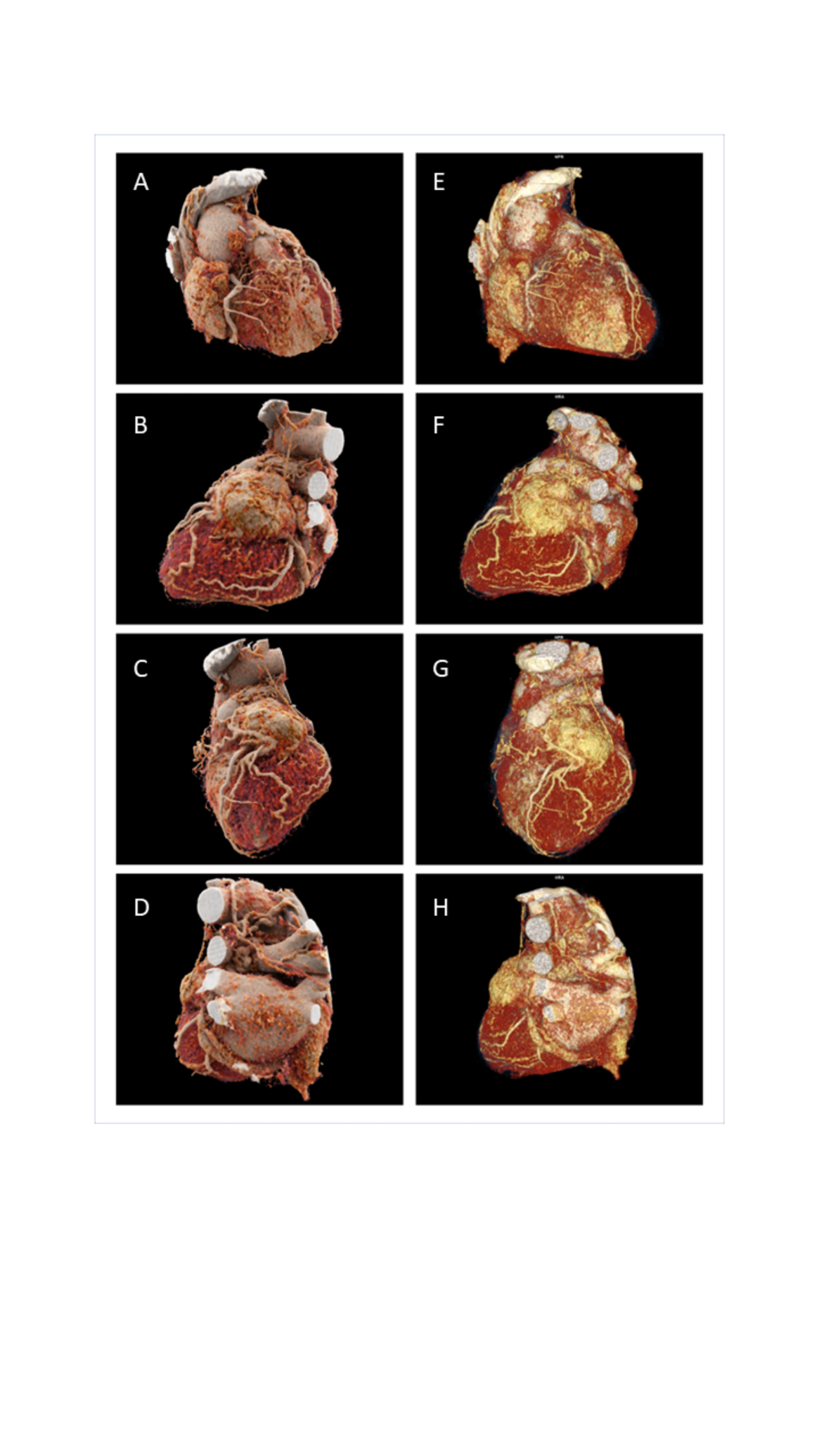


**
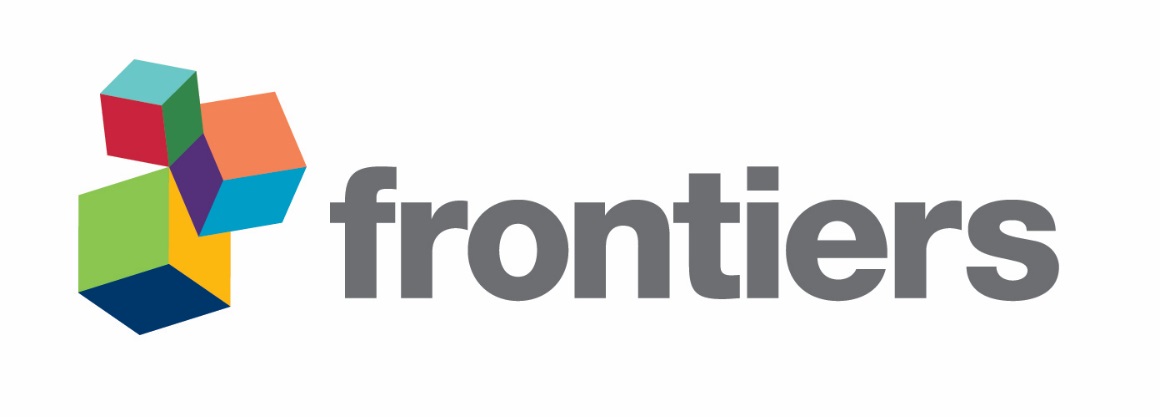
**

**Supplementary Figure 1.**

Cinematic Rendering (A-D) vs Volume Rendering (E-H) of PGL.
